# Monitoring of strength, inflammation and muscle function in allogenic stem-cell transplantation patients – a pilot study for novel biomarker and risk stratification determination

**DOI:** 10.3389/fimmu.2023.1129687

**Published:** 2023-05-15

**Authors:** Sebastian Viktor Waldemar Schulz, Daniel Alexander Bizjak, Elena Moebes, Lucas John, Verena Wais, Donald Bunjes, Elisa Sala, Jürgen Michael Steinacker, Johannes Kirsten

**Affiliations:** ^1^ Division of Sports and Rehabilitation Medicine, Center for Internal Medicine, Ulm University Hospital, Ulm, Germany; ^2^ Unit for Allogenic Blood Stem Cell and Bone Marrow Transplants, Ulm University Hospital, Clinic for Internal Medicine III, Center for Internal Medicine, Ulm, Germany

**Keywords:** allogenic stem-cell transplantation, fatigue, physical activity, inflammatory biomarkers, motor skills

## Abstract

**Background:**

Low aerobic capacity is associated with an increased mortality risk in allogenic stem-cell transplantation (alloSCT) patients, but currently used risk scores in the pre-transplantation workup are still underestimating physical activity as a prognostic factor.

**Aim:**

To examine the physical condition, muscle function, blood inflammation and training adherence of alloSCT patients during inpatient time to identify potential biomarkers associated with development of myopathy and sarcopenia.

**Methods:**

Patients undergoing alloSCT were examined at four time points (T0: before alloSCT; T_ha_: hospital admission; T1: engraftment; T2: inpatient discharge). T0 included cardiopulmonary performance, body composition, grip and knee strength, motor skill tests (One-leg stand/Tinetti/Chair-rising), blood sampling (blood cell profiling and inflammation targets (Kynurenin/high sensitivity C-reactive Protein (hsCRP)/Tumor necrosis factor alpha (TNF-alpha)/Musclin/Galectin-3) and quality of life, state of health, fatigue, muscle weakness and physical activity by questionnaires (IPAQ/BSA/SARC-F/Fatigue). At T1 and T2, blood samples, grip strength and motor skill tests were repeated. Glucocorticoid dose and daily physical activity were documented during inpatient stay.

**Results:**

26 of 35 included patients (4 females; age 55.58 ± 12.32 years; BMI 24.70 ± 3.27 kg/m^2^; VO_2peak_ 16.55 ± 4.06 ml/min/kg) could proceed to alloSCT. Grip strength and Tinetti decreased from T0 until T2, no difference in Chair-rising test, One-leg and Tandem stand. All patients engrafted after 24.9 days ± 3.9 days. HsCRP and Kynurenine increased from T0 to T1, decreased at T2. TNF-alpha (T0vsT2/T1vsT2) and Musclin (T0vsT1) decreased. At T2, Galectin-3 was higher compared to T0/T1. Correlation analysis of grip strength and inflammatory markers revealed a positive correlation with TNF-alpha at T2. 50% of patients documented physical activity and questionnaire and reported a 50%-reduction of daily endurance and strength training between T1 to T2.

**Conclusion:**

Allogeneic stem-cell transplantation is associated with immune system vulnerability due to conditioning, increased inflammation and fatigue, and loss of muscle strength and function. In addition to hsCRP, Kynurenine seems to be a reliable biomarker to monitor acute and regenerative inflammation status of alloSCT patients, while Musclin and Galectin-3 may be added to physiological assessment regarding myopathy and sarcopenia. Grip strength and daily activity level should be documented by professionals to identify risk patients early and support them with optimal (exercise) therapy.

## Introduction

For high-risk malignant hematological diseases allogeneic stem-cell transplantation (alloSCT) is so far the only potentially curative therapy option (1). The activity survey of the European Society of Blood and Marrow Transplantation (EBMT) of 2019, reported by 700 centers in 51 countries and describing the status of Hematopoietic cell transplantation (HCT) in Europe and affiliated countries, ciphers the numbers of HCT in Europe and collaborating countries with 48,512 HCT in 43,581 patients, comprising of 19,798 (41%) allogeneic and 28,714 (59%) autologous HCT (2, 3).

A high clinical importance is the risk stratification for transplantation-associated complications when selecting patients for HCT (4). Two main score systems, the hematopoietic cell transplantation-specific comorbidity index (HCT-CI) and the EBMT-DRST-Score, are currently established in order to estimate the nonrelapse mortality for patients undergoing alloSCT, thus supporting the decisional process for an alloSCT (5–8). We already showed that in addition to the HCT-Cl and EBMT risk stratification, e.g., based on comorbidities, disease stage, previous treatments, type of donor and patients’ age, performance capacity is a valuable asset of mortality prediction (9). In individuals with low aerobic capacity (AC) a more than two-fold increase of mortality could be observed (9) and therefore we proposed an improved risk stratification by a combination of muscle mass, grip strength, and AC assessment in patients undergoing alloSCT (9).

Patients who undergo alloSCT are usually admitted for several weeks, thus risking to lose muscle strength and function over time (10, 11). Furthermore patients develop acute Graft-versus-Host Disease (GVHD) in approximately 30-50% of cases (12) and chronic GVHD in 30-70% of cases (13). Glucocorticoid treatment of GVHD can lead to steroid myopathy among other side effects. Steroid myopathy is characterized by fatigue, muscle weakness and atrophy and occurs particularly in elderly people. It is caused by the direct catabolic effect of glucocorticoids. Glucocorticoids cause increased protein catabolism through increased transcription of proteolytically active enzymes and also reduce protein synthesis (14, 15). Physical activity can be effective in preventing the development of steroid myopathy (16–18). In order to establish how much activity can actually be implemented during the inpatient time, it is important to monitor the activity and document the exercises as well as monitoring muscle function by assessing motor skills and grip strength.

Aerobic as well as resistance training show many positive effects also in patients undergoing allogeneic HCT: Improvement on quality of life (19–21), endurance/aerobic capacity (22, 23), muscle strength (22–24) and functional capacity (Baumann et al., 2010; Jarden et al., 2009). Exercise also reduces fatigue/pain, anxiety, depression and aggressive or hostile behavior in this patient population (21).

In contrast to the better examined aforementioned physiological and psychological outcomes, immunological effects of exercise in HCT patients are still poorly explored. The positive effect of moderate-intensity physical activity on immune function is generally well established in healthy young people (25), and at least in non-immunocompromised individuals (26). In addition, exercise stimulates the release of stem cells with strong regenerative potential from their source of origin (e.g. bone marrow) into the bloodstream (27).

Daily exercise before and after HCT may accelerate T-cell immune reconstitution (28), although not all studies found a significant effect (29). In addition, exercise has great potential as an adjuvant for natural killer (NK) cell-based immunotherapy. It can activate NK cells to kill human leucocyte antigen (HLA)-expressing tumor cell lines that are normally resistant to lysis by NK cells (30). Thus, although exercise training shows potential as an adjunctive therapy to improve health status after allo-SCT (in particular, this lifestyle intervention may improve physical fitness and possibly immune function while attenuating fatigue), there is a need for more randomized control trials focusing specifically on GVHD (31), as most of the conducted studies are based on murine models. These studies demonstrated that a training program resulted in lower TNF-α and IL-4 levels (32). Evidence on autophagy in mice with chronic GVHD has also been shown. Autophagy is an intracellular quality control mechanism and is responsible for the degradation and recycling of damaged macromolecules and organelles. After 12 weeks of training, markers of autophagy such as autophagy-related gene 12 (Atg12) and microtubule-associated protein 1 light chain 3 alpha (LC3B) increased, but studies in human GVHD-patients confirming these observations are still missing.

In addition it is known that GVHD after transplantation is characterized by increased activation of the immune system (33), and these high, chronic levels of inflammation correlate with functional limitations and reduced physical performance (34, 35). So far, it remains unclear whether a connection can be established between increased inflammation (e.g., measured by C-reactive protein (CRP) as a classic inflammation marker) and muscular dysfunction in stem-cell transplant patients. Furthermore, biomarkers assessing muscular adaption during alloSCT therapy are only scarcely examined. Possible novel markers include the myokine Musclin. It was observed that Musclin is stimulated by activity that results in enhanced physical endurance and retarding of muscle atrophy during cancer cachexia in rodents. For this reason it is regarded as a novel biomarker for muscle function in humans (36, 37). In addition, Galectin-3 may be a useful asset to current alloSCT monitoring, as the protein plays a role in diverse biological processes such as chemoattraction, cell growth or apoptosis. It is also involved in various pathophysiological processes (e.g. heart failure, inflammation, malignant diseases) (38–40) and is proposed as a potential therapeutic target by Galectin-3 inhibitors for countering cancer progression (41). Thus, monitoring of (over-) expression of Galectin-3 may allow to prognose alloSCT severity and development. Furthermore, Kynurenine is increasingly produced in cancer patients (42, 43) and, as an immune suppressor, not only has a reinforcing influence on depressive mood (44), but also has a negative effect on anti-cancer therapy (42, 43). Exercise decreases kynurenine metabolism and protects against stress-induced depression (45). In kidney transplantation Kynurenine is already used at an early stage as a diagnostic tool for assessing post-transplantation inflammatory complications and monitoring the efficacy of therapeutic interventions (46).

The primary goal of our prospective pilot study is to investigate the effects of alloSCT inpatient time on motor skills and muscle function. We also aim specially to find inflammatory biomarkers that will give us an overview of fitness and molecular changes before and during the inpatient stay. All data were evaluated before and during the inpatient stay until discharge with regard to sport motor skills and blood variables (clinical blood profiling and new (Kynurenine, Musclin and Galectin-3) versus established biomarkers (hsCRP, Tumor Necrosis factor alpha (TNF-alpha))). In addition, physical activity on the ward was documented by the patients, previous physical activity was assessed using questionnaires and clinical parameters such as the occurrence of GVHD and administered doses of glucocorticoids were noted.

## Material and methods

35 patients were included in the period from October 2020 to June 2021. Inclusion criteria for the study were the presence of a high-risk hematological disease with indication for alloSCT and written informed consent to participate.

Exclusion criteria were, in addition to a lack of consent, the diagnosis of an acute cardiac or pulmonary disease that would have required further intensive diagnostics. This included but was not limited to unstable angina pectoris in the stress test, ischemic changes in the stress echocardiography or acute valvular vitiation.

Approval for the study was granted by the ethics committee of the University of Ulm (application no. 282/20). This trial was registered in the German register of clinical trials (DRKS00023509) and was carried out according to the declaration of Helsinki.

### Study design

Three main and one additional data collection time points were determined: T0 was the date of enrollment into the study and part of the pre-transplantation workup. These screening procedures included measurements of cardiopulmonary performance (Cycle ergometry), body composition, muscle strength and a motor skill test. Blood samples were taken and examined. In addition, participants were asked to fill out questionnaires regarding quality of life, state of health and - beginning with at hospital admission T_ha_- fatigue, muscle weakness and physical activity were documented.

The period around engraftment was defined as T1. Engraftment was defined as a leukocyte concentration >0.5 x 10^9^/L on at least three consecutive days after the transplantation. Accordingly, T1 in this study was the day on which the leukocyte concentration was above the threshold value for the first time (± 2 days). T2 was the last measurement on the day of patients discharge or the day before. At T1 and T2, blood samples were analyzed, grip strength was measured and a motor skill test was performed. During the inpatient stay, the total weekly glucocorticoid dose and daily physical activity were documented.

### Screening examinations

#### Cardiopulmonary exercise testing

All patients underwent CPET to assess their AC by measuring VO_2peak_, combined with electrocardiogram (AMEDTEC Cardiopart 12B, AMEDTEC Medizintechnik Aue GmbH, Aue, Germany) on a cycle ergometer (Lode Excalibur Sport, Lode B.V., Groningen, The Netherlands) as described in Kirsten et al. (9). A ramp wise incremental test protocol (25 W + 15 W/min) till voluntary exhaustion was used to assess VO_2peak_ during CPET utilizing a breath-by-breath metabolic analyzer (Ergostik, Geratherm, Geratal, Germany). VO_2peak_ values below 80% of the individual normative values adjusted to sex, age, body mass, and height using the Hansen/Wasserman equations (47) were rated as “low”. VO_2peak_ was defined as the highest 30-s rolling average with a respiratory exchange ratio RER ≥ 1.10. In n=48 patients who failed to reach cardiopulmonary exhaustion, ventilatory threshold 1 was assessed and results below 80% of the normative values were rated as “low” AC (47).

#### Muscle strength

Muscle strength was assessed by measuring grip strength that was measured in triplicate on both hands using a hand dynamometer (SH1003, Saehan Corp., Donghae, Korea) (48–50). The highest value was used for the classification according to age and sex related normal values from a large European population (51). This test was repeated at T1 and T2.

In addition to grip strength, the maximum strength of the right and left knee extensors was determined individually just on day T0. For this purpose, a weight of about 10% of the body weight was used in the warm-up phase. The load increased until under ten repetitions could be performed with a Borg value of at least 15-17. The one-repetition maximum (1RM) was calculated using the Brzycki formula (52, 53).

#### Anthropometry

Body composition was analyzed using bioelectrical impedance technology (InBody770, Biospace Korea, Seoul, Korea). Total muscle mass was adjusted to body height and values below 10.76 kg/m^2^ in men and below 6.76 kg/m^2^ in women were rated as pathologically low (9). The body height was measured before using a stadiometer.

#### Quality of life and sarcopenia

The mental state is evaluated using the self-report questionnaire Hospital Anxiety and Depression Scale (HADS-D - German version) (54). It is used to assess anxiety and depression in patients with physical diseases or (possibly psychogenic) physical complaints. In two independent subscales (depression and anxiety) with a total of 14 items, the psychological symptom areas most frequently mentioned for this disorder group are depicted. The test results are for orientation purposes only and do not determine a diagnosis. Scores ≤7 are considered unremarkable, scores of 8-10 are considered borderline and scores ≥11 are considered noticeable.

The SARC-F questionnaire is used for the detection of a possibly existing sarcopenia, ≥4 points indicate the presence of sarcopenia.

#### Motor skill tests

The Tinetti test can be used as an additional examination in the diagnosis of sarcopenia and to detect an increased risk of falling. The exercises to be performed include sitting and standing balance tests, trunk stability, gait pattern and coordination when raising and lowering the body from a chair. A maximum of 16 points are awarded for balance exercises and a maximum of 12 points for gait exercises. In total, a maximum of 28 points can be achieved. Patients with ≤18 points have a high risk of falling, 19-23 points a moderate risk and patients with more than 24 points have no increased risk of falling or being dependent on aids (55). Also the tandem stand and chair-rising test of the Short Physical Performance Battery (SPPB) test is used to assess coordination and balance problems (56–58). By single-leg stand each standing leg is measured once. The patient stands on one leg with the foot of the other leg resting against the knee of the standing leg. The arms are abducted. The time is measured but is stopped after a maximum of 60 seconds.

#### Daily activity and exercise documentation during inpatient time

Daily activity was assessed on T_ha_ by physical activity and sports questionnaire (BSA) and International Physical Activity Questionnaire (IPAQ) (59). While BSA referred to the past four weeks, IPAQ was based on an average week before diagnosis.

During inpatient time fatigue, duration of strength and physical aerobic activity training in minutes as well as the level of exertion during training using the Borg scale (60–62) were assessed.

There was no general sports program for every patient, but rather the implementation of offered exercise programs was reviewed.

#### Blood analysis: inflammation and cardiovascular/muscular variables

For blood profiling, 10.2 ml EDTA-anticoagulated, 4.7 ml Lithium-Heparin-anticoagulated and 7.5 ml serum blood samples (Sarstedt, Nümbrecht, Germany) were taken from a vein in the forearm. Blood sampling was conducted before the patients were physically active. Blood cell analysis was conducted by clinical standards with the DXH Coulter or the Sysmex system (Based on resistance measurement principle (impedance measurement, Coulter measurement principle), photometric measurement, differentiation in a flow cell by means of laser *via* VCSn technology (volume, conductivity, scatter)) as well as ECLIA (Roche Immunoassay Analyzer Cobas 8000, Cobas t 711 and Cobas t 511) and ELISA measurements.

#### HsCRP, kynurenine, tumor necrosis factor alpha, musclin and galectin-3

Human hsCRP (MyBioSource, San Diego, California, United States), Kynurenine (MyBioSource, San Diego, California, United States), TNF-alpha (MyBioSource, San Diego, California, United States), Musclin (Cusabio, Houston, TX, USA) and Galectin-3 concentrations (Invitrogen, Thermo Fisher Scientific, Waltham, MA, USA) were determined in serum by ELISA according to the manufacturer’s instructions.

### Statistics

Statistical analysis was performed using the SPSS software package (SPSS Statistics 21.0, IBM, Ehningen, Germany) and GraphPad PRISM (Version 9.4, La Jolla, USA). Data were tested for Gaussian distribution with Kolmogorov-Smirnov test and time point differences were tested using by One-way ANOVA with repeated measures in the case normality could be assumed. Otherwise, a Friedman’s test was used. Where data of endpoints variables were missing for at least one time point, a mixed-model approach with Tukey’s multiple comparisons test was used. Data of inpatient time in hospital were analyzed using the t-test. To test a possible interdependence of inflammation/cardiovascular function and strength, correlation analysis between Grip strength and inflammatory markers hsCRP, Kynurenine and TNF-alpha as well as the myokine Musclin and Galectin-3 were analysed with Spearman’s rank correlation coefficient. Descriptive statistics of the data are presented as mean ± standard deviation if not otherwise stated. Statistical differences were considered to be significant for values of *p ≤ 0.05, **p ≤ 0.01, ***p ≤ 0.001, ****p ≤ 0.0001.

## Results

**Figure 1:**
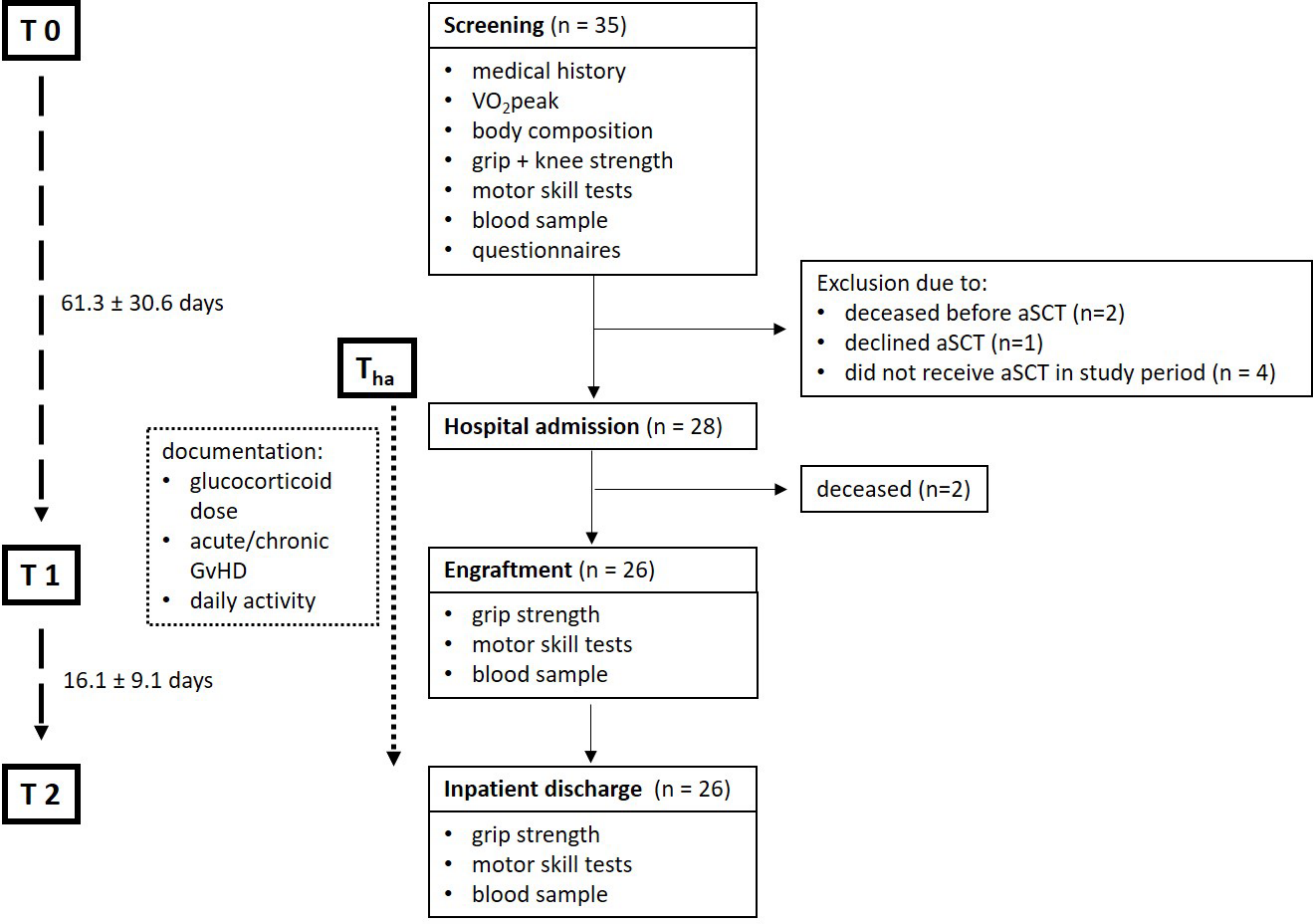
Flowchart of included participant number and data collection time points. Only data collected from participants that participated at all data collecting time points were included into the analysis (n=26): Screening (T0), hospital admission (T_ha_), engraftment (T1), inpatient discharge (T2). VO_2peak_, peak oxygen consumption; alloSCT, allogenic stem-cell transplantation; GVHD, Graft-versus-Host Disease.

In this prospective clinical study, 35 patients were included in the period from October 2020 to June 2021. Due to changes in the therapy plan or premature death before admission or during the inpatient stay, 9 patients dropped out from the study. Thus, 26 patients (4 female/22 male) were actually transplanted and could be observed as well as evaluated over the treatment course. The average time between screening and hospital admission was 36.4 days ± 29.5 (range 7 – 142) and time to engraftment from alloSCT was 24.9 days ± 3.9 (range 19 – 35). In summary in-patient time (T_ha_ – T2) was 41.0 ± 8.5 (range 31 – 74) days (**Figure 1**).

Patients´ baseline characteristics and diagnosis with time slots are represented in **Table 1**. Further information on conditioning and GVHD can be found in the **Supplementary Table S1**. In summary, 76.9% received a myeloablative, 23.0% a dose reduced and no patient a non-myeloablative conditioning.

**Table 1:** Patients’ main demographic and clinical characteristics.

		[n]	[%]
Sex	male	22	84.6%
	female	4	15.4%
	all	26	100.0%
Diagnosis	AML	12	46.2%
	ALL	2	7.7%
	MDS	2	7.7%
	OMF	8	30.8%
	CLL	2	7.7%
Number of alloSCT	1st HSCT	22	84.6%
	2st HSCT	4	15.4%
Type of HSCT	allogenic	26	100.0%
Source of donor cells	peripheral blood	26	100.0%
Origin of cells in HSCT	family	5	19.2%
	matched/mismatched unrelated donor	21	80.8%
Conditioning regimen	myeloablative	6	23.1%
	myeloablative/dose reduced	20	76.9%
HLA-match status in HSCT	HLA-matched and related	5	19.2%
	HLA-matched and unrelated	17	65.4%
	HLA-mismatched (un-/related)	4	15.4%
Graft manipulation method	unmanipulated	26	100.0%
Risk factors cardiovascular		21	80.8%
HCT-CI	0	23	88.5%
	1	3	11.5%
ECOG Status	0	19	73.1%
	1	7	26.9%
	2	0	0.0%
	3	0	0.0%
	4	0	0.0%
	5	0	0.0%
DRST-EBMT Score	0	0	0.0%
	1	1	3.8%
	2	0	0.0%
	3	5	19.2%
	4	3	11.5%
	5	9	34.6%
	6	5	19.2%
	7	0	0.0%
	8	0	0.0%
aGVHD	none	6	23,1%
	I	3	11.5%
	II	5	19.2%
	III	10	38.5%
	IV	0	0.0%
		**Mean ± SD**	**(Min - Max)**
Days from diagnosis to HSCT		970.2 ± 1637.5	(103, 5151)
Days from HSCT to aGVHD		19,6 ± 13,8	(10; 62)

The anthropometric, cardiopulmonary, strength and quality of life data are presented in **Table 2**. Ten of 22 men and no women had a pathologically low total muscle mass. HADS-questionnaire showed noticeable values in anxiety of n=21 and in depression of n=7. In the SARC-F 13 patients scored 0 points, 6 patients scored one point and 7 patients scored two points. No patient scored more than two points.

**Table 2:** Patients’ characteristics of anthropometric, cardiopulmonary, strength and quality of life at T0; n=26.

		Mean ± SD	
Age	[years]	55.6 ± 12.3	**anthropometry**
Height	[cm]	175.4 ± 8.6	
Body mass	[kg]	76.7 ± 14.9	
Body mass index	[kg/m²]	24.7 ± 3.3	
Muscle mass absolute	[kg]	32.3 ± 6.7	
Muscle mass relative	[kg/m^2^]	10.4 ± 1.4	
Body fat absolute	[kg]	17.7 ± 6.3	
Body fat relative	[%]	22.8 ± 6.0	
P_peak_	[watt]	114,5 ± 39.7	**cardiopulmonary**
VO_2_	[mL/min]	1,3 ± 0.4	
VCO_2_	[mL/min]	1,6 ± 0.5	
VO_2peak_	[mL/min/kg]	16,6 ± 4.1	
RER		1,2 ± 0.1	
HR_max_	[bpm/min]	142,5 ± 17.9	
Knee strength	[kg]	34.9 ± 11.4	**strength**
Anxiety		11.8 ± 2.3	**Quality of Life**
Depression		9.1 ± 2.1	
SARC-F		0.8 ± 0.9	
Fatigue hospital admission		2,7 ± 2,3	
Fatigue at HSCT		6,4 ± 2,3	
Fatigue inpatient discharge		2,8 ± 1,9	

P_peak_, peak power; VO_2_, oxygen consumption; VCO_2_, carbon dioxide output; VO_2peak_, peak oxygen consumption; RER, respiratory exchange rate; HR_max_, maximum heart rate; HSCT, hematopoietic stem-cell transplantation.

### Physical performance and motor skill tests

**Figure 2:**
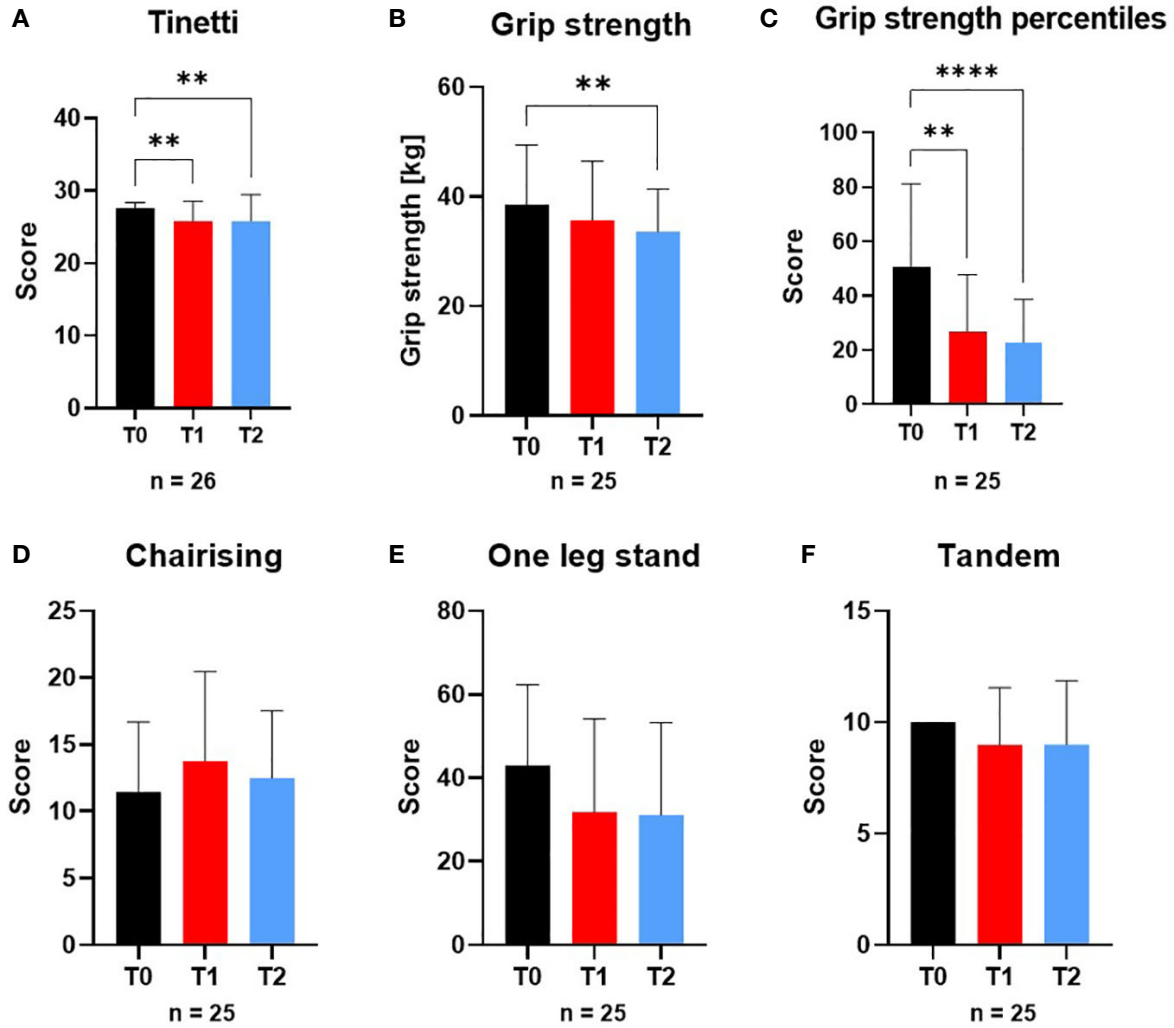
Variables of physical performance and motor skill tests at screening (T0), engraftment (T1) and inpatient release (T2). The variables of physical performance and motor skill tests **(A)** Tinetti test, **(B)** Grip strength, **(C)** Grip strength percentiles, **(D)** Chair-Rising test, **(E)** One-leg-stand-test and **(F)** Tandem-standing were affected by the conditioning phase during alloSCT. From T0 to T2 Tinetti test and Grip strength were significantly different to T0 as well as Tinetti test from T0 to T1. **p ≤ 0.01, ****p ≤ 0.0001.

Tinetti test decreased at all-time points (n=26): T0 (27.6 ± 0.8), T1 (25.8 ± 2.7; p=0.002) and T2 (25.9 ± 3.7; p=0.009). While in T0 no patient had a low or moderate risk to fall, in T1 and T2 two patients developed an increased risk of falling. Grip strength (n=25) decreased significantly at T2 (p=0.002), grip strength percentiles decreased continuously from T0 to T2 (T0 (50.8 ± 30.4), T1 (26.8 ± 20.9; p=0.003) and T2 (22.8 ± 15.8; p<0.0001)). Chair-Rising, one-leg-stand and tandem-test did not differ significantly at any time-point (**Figure 2**).

### Daily activity

The patients reported in BSA-questionnaire that they walked within four weeks prior to T0 about 42.96 ± 40.1 floors per day (n=25). In this time the duration of physical activity in daily and leisure time life was 7789.3 ± 5899.5 Metabolic Equivalent of Task (MET) min/week (n=25). Thus, 65.4% had a high activity level, 34.6% had a moderate activity level and nobody had a low activity level.

In IPAQ the participants stated with an average of physical activity of 235.9 ± 210.1 min/week (n=25) and the additional sports activity was 60.35 ± 233.5 min/week (n=25).

### Exercise documentation during inpatient time

#### Fatigue

**Figure 3:**
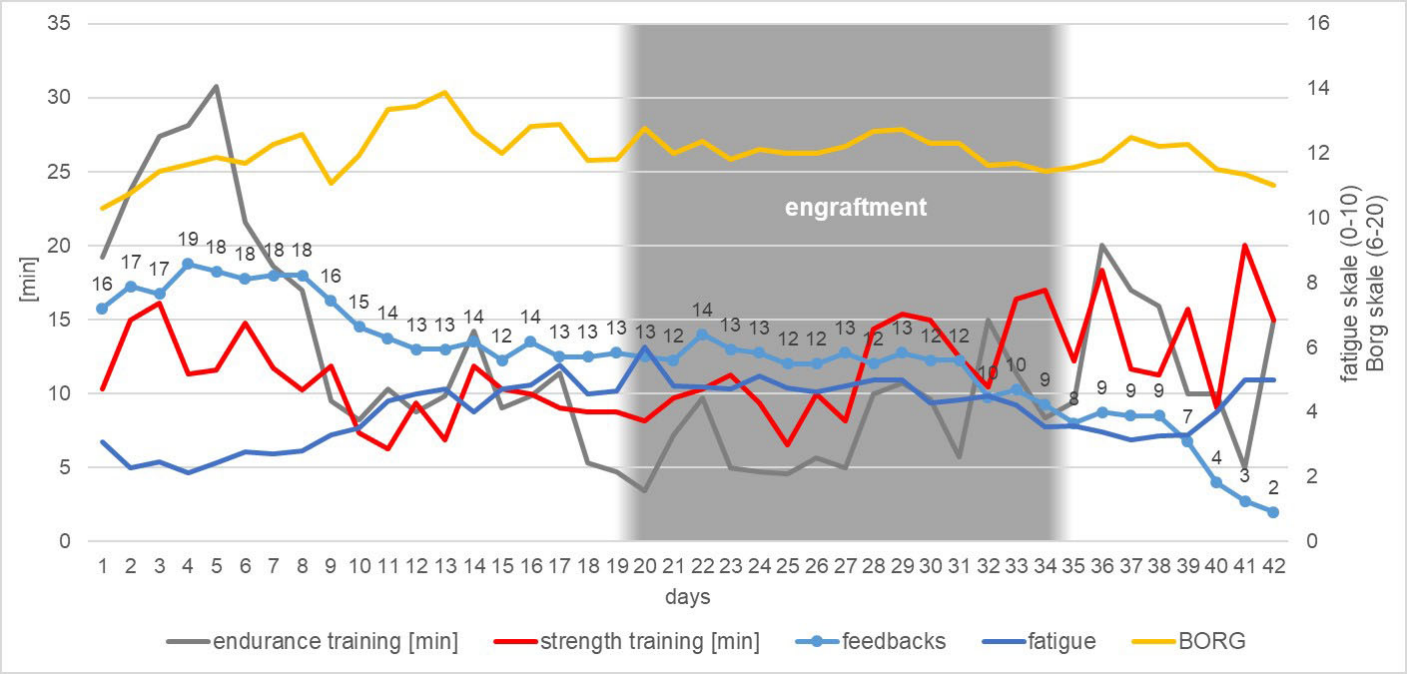
Summary of the development of the patients’ daily activity during their inpatient stay regarding endurance training, strength training, questionnaire feedbacks, general fatigue scale and exercise intensity assessed by BORG-scale (Borg, 1982).

Up to day 25 of the inpatient stay, which corresponded approximately to the time of T1, the patients (n=13) reported an average fatigue of 4.0 ± 1.2 (range 2-6). Between day 25 and the day of discharge, fatigue averaged 4.1 ± 0.7 (range 3-5) (**Figure 3**).

#### Physical aerobic activity training

Until day 25, patients reported doing an average of 14.0 ± 8.2 minutes of physical aerobic activity training per day. Overall, each patient performed an average of 348.8 ± 204.6 minutes of total physical aerobic exercise up to day 25. After day 25, significantly less physical aerobic exercise was performed: An average of 7.2 ± 7.0 minutes (p=0.02) per day until discharge, and a total of 123.2 ± 118.2 minutes (p=0.002) of physical aerobic exercise on average from day 25 until discharge (**Figure 3**).

#### Strength training

In strength training no differences in the average training time was observed (T0a–T1 = 12.11 ± 7.0 minutes per day; T1–T2 = 9.6 ± 6.3 minutes per day), but there were differences in the total time (T_ha_–T1 = 302.8 ± 174.9 minutes; T1–T2 = 163.1 ± 107.2; p=0.007) (**Figure 3**).

#### Borg

There were no significant differences in exercise intensity in the period before and after day 25. On average, up to day 25, patients rated their effort of endurance and strength training with a Borg value of 11.7 ± 2.1. From day 25 onwards, a mean Borg value of 12.3 ± 1.3 was achieved (**Figure 3**).

#### Physiotherapy

The patients received physiotherapy on 13.15 ± 6.13 days during inpatient period, i.e., on average one person was activated to exercise every third day (**Figure 3**).

#### Inflammation and glucocorticoids

We considered the whole corticosteroid doses administered during the inpatient time (e.g. corticosteroids administered during the preparative regimens or as a treatment of chemotherapy-induced nausea, as well as the steroid treatment of GVHD). The average weekly glucocorticoid dose administered to the 26 patients was 4.5 ± 3.9 mg/kg body weight in the whole group. In total, a mean of 27.9 ± 27.7 mg/kg body weight of glucocorticoids was administered to each patient during the entire inpatient stay.

Considering all patients, on average most glucocorticoids were given in the second week of hospitalization. The glucocorticoid dose administered per week in each case is shown in **Supplementary Table S2**.

### Blood variables

#### Red blood cell system

**Figure 4:**
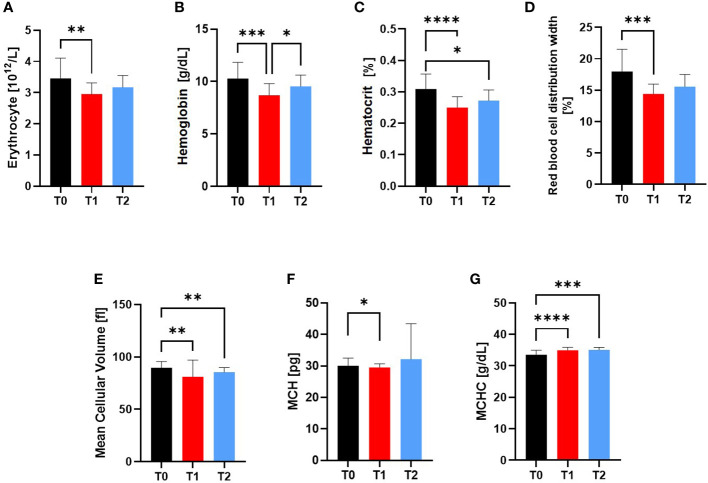
Variables of the Red blood cell system at Screening (T0), Engraftment (T1) and inpatient release (T2). The Red Blood Cell Variables **(A)** RBC, **(B)** Hemoglobin, **(C)** Hematocrit, **(D)** RDW, **(E)** MCV, **(F)** MCH and **(G)** MCHC were affected by the conditioning phase during alloSCT. Even after regeneration from T1 to inpatient release T2, the hemoglobin concentration, hematocrit, MCV and MCHC were still significantly different to T0. *p ≤ 0.05, **p ≤ 0.01, *** p ≤ 0.001, ****p ≤ 0.0001. RBC, Red Blood Cell; MCV, Mean Cellular Volume; MCH, Mean Cellular Hemoglobin; MCHC, Mean Cellular Hemoglobin Concentration; RDW, Red Blood Cell Distribution Width.

Compared to T0, red blood variables erythrocytes (p=0.0086), hemoglobin (p=0.0009), hematocrit (p<0.0001), red blood cell distribution width (RDW) (p=0.0004), mean cellular volume (MCV) (p=0.0016) and mean corpuscular hemoglobin (MCH) (p=0.0457) were still decreased at engraftment time point T1, whereas the mean corpuscular hemoglobin concentration (MCHC) increased in the alloSCT patients at T1 (p<0.0001) and T2 (p=0.0005) (**Figure 4**).

At inpatient release at T2, RBC, hemoglobin, RDW, and MCH were not significantly different to T0 anymore.

### Inflammation

#### HsCRP, kynurenine and TNF-alpha

**Figure 5:**
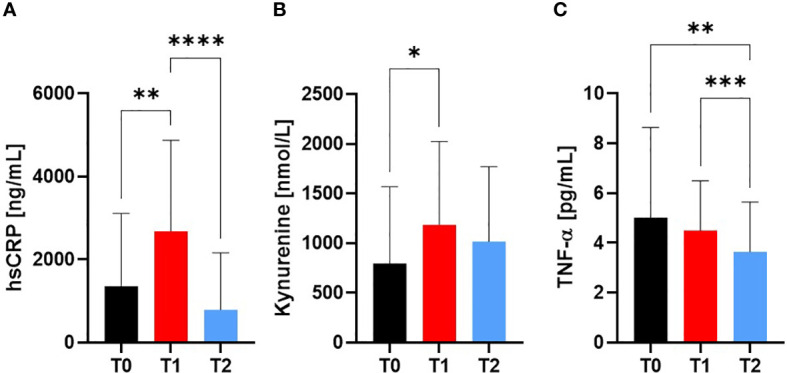
Markers for Inflammation at Screening (T0), Engraftment (T1) and inpatient release (T2). The pro-inflammatory markers **(A)** hsCRP and **(B)** Kynurenine increased from T0 to T1, whereas **(C)** TNF-alpha showed decreased values at T2 compared to T0 and T1. At T2, a reduction in pro-inflammation was observed by similar levels to baseline measurement T0. *p ≤ 0.05, **p ≤ 0.01, *** p ≤ 0.001, ****p ≤ 0.0001.

HsCRP increased from T0 (1.36 ± 1.8 µg/mL) to T1 (2.68 ± 2.2 µg/mL; p=0.0042) and decreased at T2 compared to T1 (0.79 ± 1.4 µg/mL; p<0.0001; **Figure 5A**), while there was an increased Kynurenine concentration at T1 compared to T0 (p=0.0327; **Figure 5B**). TNF-alpha decreased from T0 to T1 (p=0.0054) to T2 (p=0.0003) (5.0 ± 3.6 vs 4.5 ± 2.0 vs 3.6 ± 2.0 pg/mL, respectively; **Figure 5C**).

#### Muscular and cardiovascular health

**Figure 6:**
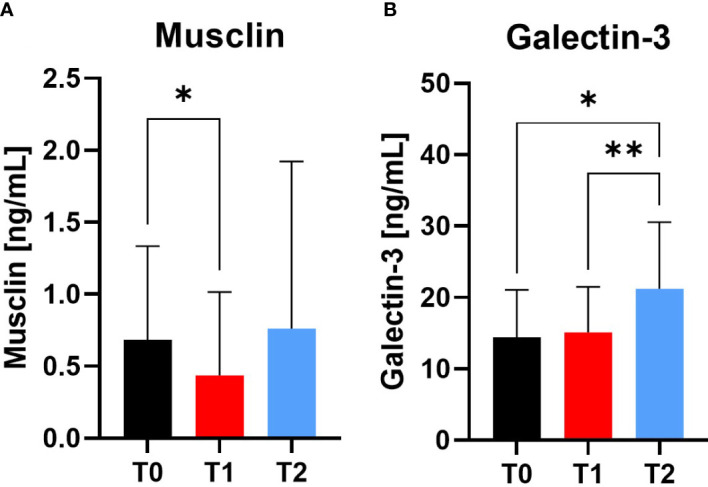
Variables of muscular and cardiovascular health at Screening (T0), Engraftment (T1) and inpatient release (T2). The myokine Musclin **(A)** decreased from T0 to T1, indicating a catabolic muscle status. In contrast, Galectin-3 **(B)** increased at T2 compared to T0 and T1. *p ≤ 0.05, **p ≤ 0.01.

The myokine Musclin decreased significantly at T1 compared to T0 (p=0.0261), whereas there was no difference at T2 observable. Galectin-3 as marker for cardiovascular health increased at T2 compared to T0 (p=0.0188) and T1 (p=0.0035) (**Figure 6**).

#### Correlation analysis of inflammatory markers and grip strength

There was a positive correlation between TNF-alpha and grip strength at T2 (r=0.41; p=0.045). No other significant association of inflammatory markers and grip strength was observed (**Supplementary Figure S1**).

## Discussion

Allogenic stem-cell transplantation includes severe treatment before, and long hospitalization time during transplantation. These phases are associated with immune system susceptibility by conditioning, increased inflammation and fatigue, with subsequent loss of muscle strength and function.

In this pilot study, our examinations underline the decline of grip strength, muscle function over time and an acute pro-inflammatory status at engraftment. Despite the offer of physical activity and physiotherapy, and although fatigue remained unaltered, alloSCT patients’ training adherence declined during their respective inpatient stay.

These observations underline the current problem of insufficient physical activity during therapy, albeit a correlation between the occurrence of sarcopenia and negative outcome in stem-cell transplant patients is already known (10). In addition, patients with sarcopenia at the time of stem-cell transplantation had higher rates of fatigue, less muscle strength, longer hospitalization time, and lower survival than patients without preexisting sarcopenia (11, 63). Unfortunately, beside all this evidence, assessment and monitoring of (pro-) inflammatory biomarkers, cardiovascular health and muscle function is still not established in the clinical routine.

The diagnosis of muscle loss/sarcopenia plays a central role in the assessment of muscular functions. In the framework of various tests, muscle strength, muscle function and motor skills are assessed (64), but the cutoff values referenced in the sarcopenia guidelines are largely derived from patient populations over 70 years of age (57, 65–67).

In our pilot study, we did not observe any distinctive sarcopenic features in the patients during screening: neither the grip strength, the chair-rising test nor the muscle mass per body surface area were conspicuous in view of the reference values from current guidelines, with the only exception observed in the SPPB test. Sarcopenia could well be present in the patients but not diagnosed due to lack of age- and sex-matched reference values. Thus, in stem-cell transplant patients, the diagnosis of sarcopenia is therefore only possible to a limited extent with the previously specified limits.

During inpatient treatment, there was a significant decrease in grip strength of nearly 5 kg on average. This corresponds to a decrease of approximately 13% relative to the baseline value at T0. Due to the steady loss of grip strength, 11 people dropped below the 20 percentile of grip strength reference values from T1 onwards and remained on this low value until T2. Sport motor tests such as the Tinetti test showed a muscle function deterioration, so that two patients developed a risk of fall. Similar results were obtained by Kramer et al., who showed a grip strength loss of 6% compared to baseline in patients one month after alloSCT. Patients who had developed acute GVHD in this study even showed a loss of 12% (68). The study by Morishita et al. also showed that grip strength and knee extensor strength decreased significantly during the inpatient stay for alloSCT, and the patients’ balance deteriorated (69). However, it remains unexplained in the studies which factors have a particular influence on the deterioration of these abilities.

### Corticosteroids and strength

As already mentioned, steroid myopathy caused by the direct catabolic effect of glucocorticoids is a common side effect during alloSCT. Besides the general wide range and individual risk factors (12), myopathy can contribute to the development of GVHD (70). We examined our cohort only during the inpatient stay, so we cannot draw any conclusion about the connection between GVHD and physical activity, as chronic GVHD is normally diagnosed up to day 100 (13) and longer assessment would be needed in our subjects.

Our patients received an average glucocorticoid dose of 4.5 ± 3.9 mg/kg body weight per week and a mean of 27.9 ± 27.7 mg/kg body weight during the entire inpatient stay. Therapy of choice for acute GVHD includes high-dose glucocorticoids (71, 72) and the German Working Group for Bone Marrow and Blood Stem-cell Transplantation recommends (in addition to other immunosuppressive medications) 1-2 mg of prednisolone/kg/body weight/day for up to seven days, then a dose reduction of 20% every 5 days (73). Regarding our mean inpatient stay of around 77 days, the administered dose thus fell well into the guideline recommendations.

Morishita et al. examined 113 patients who received alloSCT and observed correlations between the dose of steroids administered with a decrease in strength of the hand and knee extensors (74). This result could not be replicated in our study where the grip strength significantly decreased compared to baseline measurements, regardless of steroid dosage. One explanation for this could be that the patients in the study by Morishita et al. received an average of 57.6 mg/kg glucocorticoids, which is almost twice the dose received by patients in our study. Thus, the total dose per kilogram of body weight during the observation period is larger in Morishita et al. with presumably higher impact for developing myopathic symptoms

Unfortunately, there was a reduction in the physical activity level in our alloSCT patients after engraftment, nearly after two weeks of inpatient stay. The daily time of endurance training decreased from 14 min/day before engraftment to 7 min/day after engraftment. Strength training also decreased steadily, but from 12 to 10 min/day, thus only the total time of strength training was significantly different. The cause of this development can be explained by the weakened immune system. A possible reason that our patients did not concomitantly report an increased load intensity during training with regard to the Borg scale could be the low daily resistances or intensities when they are active or exercise. It could be speculated that the activity time would drop maybe even lower if there would not be supervised physiotherapy nearly every three days during inpatient stay.

One explanation for the decreased activity time would normally have been increased fatigue, but this was not reported in this case. According to the literature, one would have expected a higher fatigue towards the end of the inpatient stay due to the reduced physical activity (75, 76), but this was not supported by our questionnaire evaluation with similar fatigue during the whole stay.

The evidence of beneficial effects of training – especially for alloSCT patients – is growing. Baumann et al. showed that patients receiving alloSCT can reduce strength losses of the knee extensors by increased training (23) with probably positive outcomes for gait speed and falling risk reduction. Furthermore, a review by Wiskemann et al. reported significant improvement by exercise interventions for physical performance, quality of life and fatigue as well as faster recurrence of immune cells or reduced severity of therapy-related side effects can be estimated. (77). Hence, it seems advisable to lay an additional focus of treatment before, during and after the treatment to reduce alloSCT side effects and reduce hospitalization time.

Unfortunately, the independently performed training documentation by the patients in our study was inadequately accomplished in most cases. Accordingly, the results of the sports documentation can only be assessed to a limited extent. In order to achieve more objectivity, the number of days on which the patients received physiotherapy was also recorded. As a result, this showed that a higher number and more working hours for physiotherapists are necessary to avoid the negative consequences of a lack of supervised exercise training for patients.

### Blood cells

The immunosuppressive effect of alloSCT treatment (including a conditioning phase with aplasia) and glucocorticoids can have an immense impact on physical abilities and therapy outcome. Patients receiving alloSCT are in a special situation: their hematopoietic system has to be specifically eliminated and replaced by donor stem-cells due to the underlying hematological disease. In this case, the immune system must re-establish itself. Immune cells comprise approximately 2-6% of the cells in healthy skeletal muscle. Evidence exists that (chronically) elevated inflammatory markers are associated with the development of sarcopenia (35, 78–80) and that immune cells are important for the maintenance of skeletal muscle function and muscle plasticity (81). A randomized control trial with allogeneic bone marrow transplant patients was able to increase the total lymphocyte count by 40.9 cells/μl through a series of bed exercises (30 minutes daily over a period of 6 weeks). The non-exercising control group of the same study showed a decrease of 640.7 cells/μl) without affecting the composition of CD4+ and CD8+ T-cell subsets (28). Thus, fluorescence activated cell sorting (FACS) analysis to assess immune cell subgroups during alloSCT treatment and monitoring might be added to the clinical routine. Unfortunately, as was also the case in the presented study, the required blood volume, time and expertise for clinical FACS analysis is seldom available and leads to potential important missing information.

### Inflammation: HsCRP and KYN

The association between inflammatory status on the decrease in muscular function in alloSCT patients, especially grip strength and sport motor skills, have already been mentioned. To assess the inflammatory status, the changes of the highly sensitive C-reactive protein (hsCRP), Kynurenine and TNF-alpha were examined in this study. CRP is an acute phase protein produced in the liver in response to inflammation or tumors, and elevated CRP levels before stem-cell transplantation correlate with a reduced probability of survival (82).

Beside the known correlation of elevated CRP levels before stem-cell transplantation with a reduced probability of survival (82), hsCRP can be used for the diagnosis of acute infections and partly for chronic inflammation - such as vasculitis - (83). We observed both an increase in hsCRP and Kynurenine at engraftment compared to baseline measurements, indicating the still high susceptibility of patients after alloSCT. This may be aggravated by the interdependence between severe treatment, increased pro-inflammation and declining physical performance capacity during inpatient stay. In addition to the well-known and established CRP, growing evidence indicate Kynurenine as a reliable biomarker to monitor disease status. Kynurenine is a metabolite during tryptophan metabolism synthesized mainly by the Indoleamine 2,3-Dioxygenase (IDO). Increased IDO activity, on the other hand, is associated with increased mortality (84), while an increase in Kynurenine levels is seen in older adults with reduced physical activity (85) as well as in the context of GVHD-related inflammation (86). Several studies report an association between elevated inflammatory markers such as CRP or Kynurenine and increased fatigue (87–89). The concentration of these markers can be reduced with training (therapy) (90–93) and it was shown that strength and endurance training can reduce fatigue during a hospitalization for alloSCT (75, 76).

Our observed increased Kynurenine levels at engraftment with subsequent baseline values at inpatient release show that Kynurenine may be a valuable addition to hsCRP to examine the acute inflammatory status of alloSCT. Furthermore, it was shown by Westbrook et al. that elevated levels of Kynurenine were associated with increased inflammatory cytokines, decreased grip strength, and decreased gait speed in humans (94). However, it remains problematic in this context that there exists no standardized measurement procedure for Kynurenine in clinical routine, which complicates the comparison of study results. In addition, we could not show an interdependence between hsCRP or Kynurenine with grip strength, so a direct link between these inflammatory markers and strength decline appears unlikely.

The biomarker panel of CRP and Kynurenine could be complemented by TNF-alpha measurements during alloSCT. TNF-α, formerly only seen as pro-inflammatory cytokine, is now regarded as a pleiotropic molecule with anti-inflammatory and immunomodulatory effects (95). Increasing TNF-alpha plays a key role in aGVHD (96) by preceding the onset of aGVHD, before peaking at the time of its development (97–99). It can also be used for the discrimination of patients with and without GVHD, the prediction of survival as well as the estimation of therapy efficacy are reliable application fields of TNF-alpha measurements (99–101). We observed decreased TNF-alpha already at T1, which further decreased at inpatient release T2. As mentioned by the colleagues Kitko et al, TNF-alpha cannot sufficiently be used as only biomarker for the prediction of the development of GVHD (96), but the combination with hsCRP and Kynurenine seems promising.

For a more sensitive view of muscle wasting during alloSCT, we examined the development of the novel biomarkers Musclin and Galectin-3 during the inpatient stay. The synthesis of the myokine Musclin is up-regulated in skeletal muscle in response to physical activity and subsequently secreted into the systemic circulation promoting skeletal muscle mitochondrial biogenesis and exercise endurance. As it was observed that Musclin may be able to alleviate cancer-induced muscle wasting during cachexia, which is characterized by systemic inflammation through elevated levels of pro-inflammatory cytokines such as TNF-alpha, it might serve as a novel marker for cachexia development and potential therapeutic target. We observed decreased levels of Musclin at engraftment, which indicates the decline in muscle synthesis during the inpatient stay and is in line with our measured declining grip strength, although no direct correlation could be observed. Most studies examining the mechanistic effect of Musclin have been determined in rodents, so further data are needed to finally elucidate the monitoring value in humans (36).

In contrast, Galectin-3 as marker for cardiovascular disease, showed highest values at inpatient release. In previous studies, this molecule was upregulated in human cancer development and progression (102). Thus, a recent study by Caputo et al. suggested Galectin-3 as a potential therapeutic target especially in the early phases of prostate cancer progression and metastasis (41). In addition, our observed values may indicate Galectin-3 as potential long-term biomarker of apoptosis/neogenesis during alloSCT therapy and cardiovascular health status due to its additional involvement in exercise and stress induced damage (103). But in how far these observations can be transferred to alloSCT and subsequently transplantation risk and health monitoring, has still to be evaluated in further controlled clinical studies.

### Limitations

In this study, motor skill skills were monitored using grip strength measurements as well as the Tinetti test, the chair-rising test, and various balance exercises during the course.

At T0, physical status was also assessed using a bioimpedance analysis, muscle strength measurements of the knee extensors, and other sport motor tests such as the SPPB test. For a more precise assessment of the progression, a renewed measurement of the body composition and muscle strength measurement of the knee extensors would have offered more information but was not possible due to the lack of equipment on the ward and for possible safety reasons (possible fall with dislocation of the catheter) as well as due to the expected false high results (the patients would have to additionally move an infusion stand in contrast to T0). Finally, in this study, all patients reported being very active before their diagnosis with no diagnoses low activity level before study start.

The above pre-transplant screening with assessment of sarcopenia was performed in about 2 hours and assessed by sports physicians. Additional resources are needed to introduce such procedures in transplant centers, but the benefit seems given the expenses of peri-transplant complications very valuable.

## Conclusion

In order to find out whether physical activity during the inpatient stay or in the period before transplantation has a protective effect against grip strength loss and sarcopenia, group-wise comparisons should be performed. For this purpose, one group should receive more intensive care in terms of additional training interventions. The feasibility has already been demonstrated in several small studies (104–106). This could also create larger differences e.g. to motor skills or markers of inflammation, document physical activity more reliably, and better demonstrate any effects that may exist.

Future studies should therefore definitely consider other factors such as diet, a genetic predisposition or the influence of other drugs beside corticosteroids. In order to be able to identify patients with an increased risk for the decline of sport motor skills at an early stage, further screening parameters should be investigated during the course. These include, for example, muscle mass analysis at all three measurement time points, knee extensor strength during the course to better assess the lower extremity as well, and, if necessary, repeated cardiopulmonary exercise testing at the time of inpatient discharge. To improve the outcome and quality of life of this fragile patient cohort additional studies and supportive training systems for discharged patients should be developed.

Integrating the pre-transplant screening with assessment of sarcopenia as a routine pre-transplant examination can help to create an individual and appropriate training program for each patient. On the one hand, this avoids that cancer patients with a poor performance status and a high disease burden are not even registered for training. On the other hand, the patients are sensitized at an early stage to the importance of physical performance, which must be maintained as well as possible or first even improved in the further course of treatment.

As a next step, prospective multi-center studies with larger samples are urgently needed, focusing in particular on patients with low performance status. We are sure that an improved offer of sports therapy can further reduce mortality in general.

## Data availability statement

The original contributions presented in the study are included in the article/**Supplementary Material**. Further inquiries can be directed to the corresponding author.

## Ethics statement

The studies involving human participants were reviewed and approved by ethics committee of the University of Ulm (application no. 282/20). The patients/participants provided their written informed consent to participate in this study.

## Author contributions

SS and DB participated in the research design, data analysis and writing the paper. EM and ES participated in the writing of the paper. EM, LJ, VW, DB and ES participated in the performance of the research. JS, JK and ES participated in proofreading the manuscript draft. JK was the principal investigator and the supervisor for this study. All authors contributed to the article and approved the submitted version.
