# Supplementary material for: Monitoring of strength, inflammation and muscle function in allogenic stem-cell transplantation patients – a pilot study for novel biomarker and risk stratification determination
Source: Front Immunol. 2023 May 15;14:1129687. doi: 10.3389/fimmu.2023.1129687 (PMC10225503; doi:10.3389/fimmu.2023.1129687)
Supplement: Supplementary file 1 [file DataSheet_1.docx]

Supplementary Table S1: Conditioning and Graft-versus-Host Disease (GVHD) prophylaxis of included patients.

|  | **[n]** | **[%]** |  |
| --- | --- | --- | --- |
| Flu/ATG/Carmustin/Melphalan | 7 | 26.9 | Conditioning |
| Flu/ATG/Treosulfan | 6 | 23.1 |  |
| Flu/ATG/Tresulfan/Amsakrin/Cytarabin | 2 | 7.7 |  |
| Flu/ATG/Busulfan/Cyclophosphamid | 2 | 7.7 |  |
| Flu/BCNU/Tiotepa/ATG | 1 | 3.8 |  |
| Flu/Treosulfan/Amsakrin/Cytarabin | 1 | 3.8 |  |
| Flu/Thiotepa/ATG | 1 | 3.8 |  |
| Flu/Thiotepa/Busulfex | 1 | 3.8 |  |
| Flu/Thiotepa/ATG/Busulfex | 1 | 3.8 |  |
| Flu/BCNU/Melphalan | 1 | 3.8 |  |
| Flu/ATG/Tresulfan/TBI | 1 | 3.8 |  |
| Flu/ATG/Tresulfan | 1 | 3.8 |  |
| ATG/TBI/Cyclophosphamid | 1 | 3.8 |  |
| Tac/Myfortic | 11 | 34.6 | GVHD prophylaxis |
| Tac/MPA | 7 | 26.9 |  |
| Tac/MMF | 2 | 7.7 |  |
| Tac | 2 | 7.7 |  |
| MPA/CSA | 1 | 3.8 |  |
| Myfortic/CSA | 1 | 3.8 |  |
| None | 1 | 3.8 |  |
| Missing | 1 | 3.8 |  |
| FLU: Fludarabin; BCNU: Bis-Chlorethyl-Nitrosourea; ATG: Antithymozytenglobulin; TBI: Total body irradiation; Tac: Tacrolismus; MPA: Mycophenolat; MMF: Mycophenolat mofetil; CSA: Cyclosporin A; GVHD: Graft-versus-Host-Disease. | | | |
