# Supplementary material for: Monitoring of strength, inflammation and muscle function in allogenic stem-cell transplantation patients – a pilot study for novel biomarker and risk stratification determination
Source: Front Immunol. 2023 May 15;14:1129687. doi: 10.3389/fimmu.2023.1129687 (PMC10225503; doi:10.3389/fimmu.2023.1129687)
Supplement: Supplementary file 2 [file DataSheet_2.docx]

**Supplementary Table S2: Mean weekly glucocorticoid dose of all included patients.** Shown is the mean glucocorticoid dose administered per week in milligrams during the inpatient stay.

|  | **Range**  [mg] | **Mean ±SD**  [mg] |
| --- | --- | --- |
| **Week 1** (n=26) | 0 - 536 | 62.8 ± 138.4 |
| **Week 2** (n=26) | 0 - 4496 | 971.4 ± 898.1 |
| **Week 3** (n=25) | 0 - 2838 | 228.3 ± 567.7 |
| **Week 4 (**n=23) | 0 - 758 | 175.0 ± 216.9 |
| **Week 5** (n=22) | 0 - 1150 | 309.1 ± 377.0 |
| **Week 6** (n=15) | 0 - 980 | 275.3 ± 271.2 |
| **Week 7** (n=8) | 6 - 1153 | 314.1 ± 431.7 |
| **Week 8** (n=4) | 27 - 1063 | 347.5 ± 482.2 |
| **Week 9** (n=1) | - | 1063.0 |
| **Week 10** (n=1) | - | 830.0 |
| **Week 11** (n=1) | - | 631.0 |
