# Supplementary material for: Monitoring of strength, inflammation and muscle function in allogenic stem-cell transplantation patients – a pilot study for novel biomarker and risk stratification determination
Source: Front Immunol. 2023 May 15;14:1129687. doi: 10.3389/fimmu.2023.1129687 (PMC10225503; doi:10.3389/fimmu.2023.1129687)
Supplement: Supplementary file 3 [file Image_1.pdf]

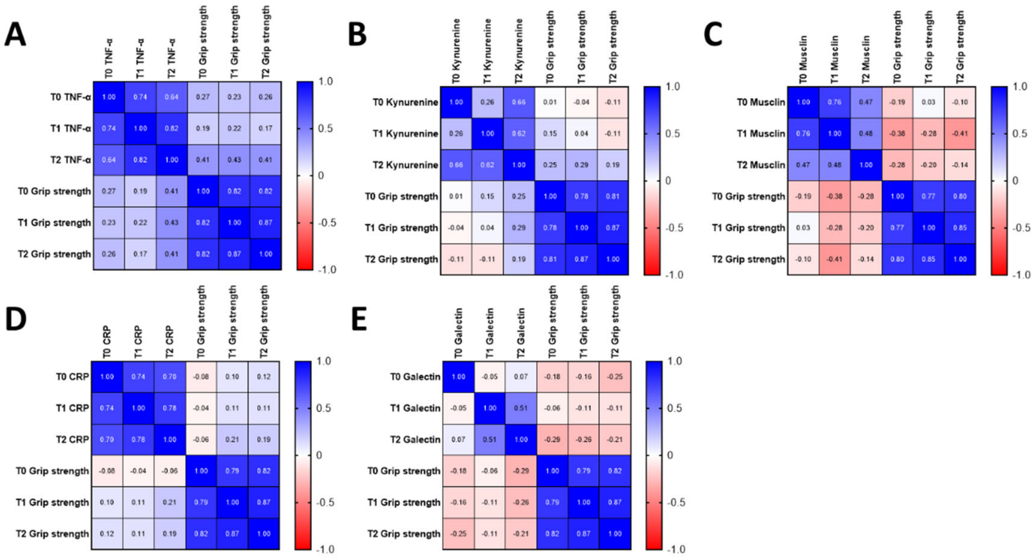

Supplementary Figure S1. Correlation analysis of inflammatory markes and grip strength at Screening (T0), Engraftment (T1) and inpatient release (T2). (A) TNF-alpha, (B) Kynurenin, (C) Musclin, (D) hsCRP and (E) Galectin were affected by the conditioning phase during alloSCT. The only significant association was at T2 between TNF-alpha and grip strength with p=0.045.
